# MMP9 SNP and MMP SNP–SNP interactions increase the risk for ischemic stroke in the Han Hakka population

**DOI:** 10.1002/brb3.2473

**Published:** 2022-01-05

**Authors:** Daofeng Fan, Chong Zheng, Wenbao Wu, Yinjuan Chen, Dongping Chen, Xiaohong Hu, Chaoxiong Shen, Mingsheng Chen, Rongtong Li, Yangui Chen

**Affiliations:** ^1^ Department of Neurology Longyan First Affiliated Hospital of Fujian Medical University, Longyan Fijian China; ^2^ Department of Acupuncture and Moxibustion Longyan First Affiliated Hospital of Fujian Medical University, Longyan Fijian China

**Keywords:** arteriosclerosis, GMDR, ischemic stroke, *MMP‐12*, *MMP‐9*, polymorphism

## Abstract

**Objectives:**

To investigate the association of eight variants of four matrix metalloproteinase (MMP) genes with ischemic stroke (IS) and whether interactions among these single nucleotide polymorphisms (SNPs) increases the risk of IS.

**Methods:**

Among 547 patients with ischemic stroke and 350 controls, matrix‐assisted laser desorption/ionization time of flight mass spectrometry was used to examine eight variants arising from four different genes, including *MMP‐1* (rs1799750), *MMP‐2* (rs243865, rs2285053, rs2241145), *MMP‐9* (rs17576), and *MMP‐12* (rs660599, rs2276109, and rs652438). Gene–gene interactions were employed using generalized multifactor dimensionality reduction (GMDR) methods.

**Results:**

The frequency of rs17576 was significantly higher in IS patients than in controls (*p *= .033). Logistic regression analysis revealed the AG and GG genotypes of rs17576 to be associated with a higher risk for IS, with the odds ratio and 95% confidence interval being 2.490 (1.251–4.959) and 2.494 (1.274–4.886), respectively. GMDR analysis showed a significant SNP‐SNP interaction between rs17576 and rs660599 (the testing balanced accuracy was 53.70% and cross‐validation consistency was 8/10, *p *= .0107). Logistic regression analysis showed the interaction between rs17576 and rs660599 to be an independent risk factor for IS with an odds ratio of 1.568 and a 95% confidence interval of 1.152–2.135.

**Conclusion:**

An *MMP‐9* rs17576 polymorphism is associated with increased IS risk in the Han Hakka population and interaction between *MMP‐9* rs17576 and *MMP‐12* rs660599 is associated with increased IS risk as well.

## Abstract 2

This study shows that the presence of one *MMP9* SNP and one *MMP* SNP‐SNP interaction increase the risk for ischemic stroke in the Han Hakka population. These findings uncover gene–gene interactions that may provide therapeutic targets.

## 1 INTRODUCTION

Ischemic stroke (IS) accounts for the majority of all strokes, and its high morbidity, disability, and mortality have seriously threatened human health (Feigin et al., 2018; Zhou et al., 2019). Epidemiological studies have shown that IS has become a major disease in China, making it one of the most important diseases leading to disability (Chao et al., 2021; Wan et al., 2021; Wang et al., 2020). The main mechanism of IS is cerebral vascular obstruction caused by atherosclerosis. Although risk factors are actively controlled, the occurrence of IS is still on the rise. Previous studies have shown that gene polymorphisms play an important role in atherosclerotic IS (Malik et al., 2018). In recent years, people have become increasingly interested in the study of matrix metalloproteinases (MMPs) and their relationship with the pathogenesis of atherosclerotic cerebrovascular disease (Chehaibi et al., 2014; Li et al., 2021; Wang & Khalil, 2018).

MMPs are a multigene family of extracellular zinc‐ and calcium‐dependent endopeptidases, which play an important pathological role in the degradation of extracellular matrix (ECM) (Abilleira et al., 2006; Chang et al., 2016; Hooper, 1994) in IS. The degradation of arterial ECM proteins is a critical step in the development of atherosclerosis (Fujimoto et al., 2008). Moreover, MMPs can digest the components of fibrous plaque caps, which leads to structural damage and accelerates plaque rupture, giving rise to plaque instability (Galis et al., 1994; Ohshima et al., 2010; Schäfers et al., 2010). Furthermore, MMPs mediate many biological and pathological processes during and after cerebral ischemic injury (Su et al., 2005; Yi et al., 2019; Yi et al., 2019). Therefore, matrix metalloproteinases play an important role in atherosclerotic IS. Although there have been many studies on *MMP* gene polymorphisms in patients with IS, the results have been controversial and ambiguous (Chehaibi et al., 2014; Djurić et al., 2012; Nie et al., 2014; Sheikhvatan et al., 2018; Zhang et al., 2015). Dan Wen et al. (2014) showed that *MMP‐1*‐1607 1G/2G and *MMP‐3*‐1612 5A/6A were risk factors for IS, while *MMP‐9*‐1562C/T was not associated with IS through meta‐analysis. Guoqian Zhang et al. showed, when a subgroup analysis by ethnicity and Hardy–Weinberg equilibrium (HWE) was performed, that *MMP‐12*‐82 A/G gene polymorphisms may be a risk factor for IS in Europe. In Africa, the presence of *MMP‐1*‐1607 1G/2G and *MMP‐12*‐82 A/G was also correlated with a significant increase in IS (Zhang et al., 2018). Recently, Shubham Misra et al. conducted a meta‐analysis of 29 studies suggesting that *MMP‐9* (−1562C/T) and *MMP‐12* (−1082 A/G) gene polymorphisms could be risk factors for IS while *MMP‐1* (−1607 1G/2G), *MMP‐2* (−1306C/T) & (−735C/T), and *MMP‐3* (−1612 5A/6A) have no association with the risk of causing IS (Misra et al., 2018). These results are inconsistent, and most scholars currently believe that race and environment are the differentiators. No known studies of *MMP* gene polymorphisms in the Han Hakka population exist, making this study a necessity.

Previously, many studies on gene polymorphisms in IS have mainly focused on single gene polymorphisms, and only a few studies on gene–gene interactions are to be found. Russian scholars have shown that MMPs can be used as regulatory targets of various genes, such as rs4322086 of *RASEF*, rs11556924 of *ZC3HC1*, rs899997 of *SLCO1B1*, and rs12449964 of *PEMT*. A synergistic effect between certain genes can increase the occurrence of cerebral infarction (Polonikov et al., 2019). Khouloud Chehaibi et al. advises to be aware of joint effects or haplotypes of MMP polymorphisms as they are stronger than the individual effect of each polymorphism (Chehaibi et al., 2014). Furthermore, Yi Xingyang et al. showed the interaction of *MMP‐9* gene polymorphisms plays an important role in the damage of the blood brain barrier (BBB) in cerebral infarction (Yi et al., 2019). Therefore, it is believed that gene–gene interactions may play an important role in the occurrence and development of IS. Currently, only a few studies on *MMP* SNP–SNP interactions exist and there are no relevant studies of the Han Hakka population. Therefore, it is necessary to analyze *MMP* gene polymorphism interactions in the Han Hakka population.

This is a case‐control study investigating whether eight SNPs of four different *MMP* genes influence the risk of IS in the Han Hakka population in Western Fujian, China.

## 2 MATERIALS AND METHODS

### 2.1 Ethics statement

This study was approved by the ethics committee of the Longyan First Affiliated Hospital of Fujian Medical University (No:2017‐013), in compliance with the Declaration of Helsinki. Each of the participants provided written, informed consent before participating in this study.

### 2.2 Study populations

The study population was comprised of 547 patients with IS and 350 healthy controls in the Hakka population in Western Fujian, China. According to the trial of ORG 10172 in the acute stroke treatment classification system (Adams et al., 1993), patients with atherothrombosis were enrolled. From December 2018 to September 2020, data were consecutively collected on 547 patients that were hospitalized in Longyan First Hospital for IS. The patients with IS were all admitted to the hospital within 72 h after the onset of symptoms, had focal neurological deficit symptoms, symptoms persisting for more than 24 h, and were confirmed by brain computed tomography (CT) as well as magnetic resonance imaging (MRI). All enrolled patients were Hakka people residing in Western Fujian.

Exclusion criteria were: (1) patients with other types of IS; (2) patients with familial IS; (3) patients with transient ischemic attacks or intracranial hemorrhage; (4) patients who were unwilling to participate in the trial; (5) patients with tumors, thyroid diseases, blood system diseases, arthritis, immune related diseases, infection, severe heart disease, severe kidney disease, and severe liver disease.

A total of 350 Hakka participants undergoing physical examination were selected as the control group during the same period in our hospital. All controls had no previous family history of stroke as confirmed by CT, MRI, and medical history. The participants enrolled in the study as controls were free of tumors, thyroid diseases, blood system diseases, immune related diseases, infection, severe heart disease, kidney disease, and liver disease.

The demographic data and risk factors were recorded in detail, including age, gender, smoking status, drinking habits, hypertension, diabetes, total cholesterol (TC), low‐density lipoprotein (LDL), high‐density lipoprotein (HDL), triglycerides (TG), and homocysteine (HCY) levels. All data were registered and kept confidentially by two doctors in the department (Dongping Chen and Yinjuan Chen, Department of Neurology, Longyan First Affiliated Hospital of Fujian Medical University).

### 2.3 Genotyping

#### 2.3.1 Genome and SNPs selection

SNPs were selected according to the following criteria: (1) the SNP had been assessed in previous research; (2) the Human SNP of each gene was newly registered in the National Center for Biotechnology Information (NCBI) database (http://www.ncbi.nlm.nih.gov/snp); (3) the SNP was logged in the human Hap Map database to locate tagged SNPs (http://www.hapmap.org/), or SNPs that identify particular haplotypes; (4) the minimum allele frequency (MAF) was greater than 0.05 in the Hap Map. With these criteria, eight variants were selected, including *MMP‐1* (rs1799750), *MMP‐2* (rs243865, rs2285053, and rs2241145), *MMP‐9* (rs17576), and *MMP‐12* (rs660599, rs2276109, and rs652438).

### 2.4 Primer synthesis

Multiple polymerase chain reaction primers were designed for the qualified SNPs of *MMP‐1, 2, 9*, and *12* using the gene library GenBank (http://www.ncbi.nlm.nih.gov/omim/) and Mass Array assay design 2.0 software by Sangon Biotech Co, Ltd, Shanghai, China (Table 1).

**TABLE 1:** Amplification and extension primers used in this study

SNPs	Forward primer and reverse primer (5′−3′)	Extension primer (5′−3′)
rs1799750	F:ACGTTGGATGTTCTTTCTGCGTCAAGACTG	GATTGATTTGAGATAAGTCATATC
	F:ACGTTGGATGGTTATGCCACTTAGATGAGG	
rs243865	F:ACGTTGGATGACATTCCCCATATTCCCCAC	CCCCACCCAGCACTC
	F:ACGTTGGATGCCTGGAAGAAGTGACTTCTG	
rs2285053	F:ACGTTGGATGTATCTCATCCTGTGACCGAG	ACCGAGAATGCGGAC
	F:ACGTTGGATGAGAGCGACTCCATCTTGAAC	
rs2241145	R:ACGTTGGATGTTCCCTGGTTATCCCAATCC	TATTGACACCTGGCACA
	R:ACGTTGGATGTGAGGCAGGGTTCTAGAAGC	
rs17576	F:ACGTTGGATGTATAATGTGCTGTCTCCGCC	GCCCCAGGACTCTACACCC
	F:ACGTTGGATGAGGGTTTCCCATCAGCATTG	
rs660599	R:ACGTTGGATGACAGCTCCCAATCCATGAGG	AATGTAAGCTCTTGTCTCTT
	R:ACGTTGGATGGAAACAAAAGTGATCCTCGG	
rs2276109	R:ACGTTGGATGTTGAGATAGATCAAGGGATG	TGTCAAGGGATGATATCAACT
	R:ACGTTGGATGGTCCGGGTTCTGTGAATATG	
rs652438	F:ACGTTGGATGCTCTTGGGATAATTTGGCTC	TTTGGCTCTGGTCTTAAA
	F:ACGTTGGATGCCCTATTTCTTTGTCTTCAC	

Abbreviations: F, forward primer; R, reverse primer; SNPs, SNPs.

### 2.5 SNP detection

Blood samples (5 ml, arm vein) from both patients and controls were drawn into sterile tubes containing sodium citrate and were stored at −80°C. DNA extraction and *MMP‐1, 2, 9, 12* SNP detection was completed by Sangon Biotech Co, Ltd, Shanghai, China. An Axyprep‐96 whole blood genomic DNA Kit (AXYGEN company) was used to extract DNA, and was separated by 0.8% agarose gel electrophoresis. The DNA was concentrated at 5 mg/L and stored at −80°C. SNPs of the eight variants of *MMP‐1, 2, 9*, and *12* were detected using the matrix‐assisted laser desorption/ionization‐time of flight mass spectrometrymethod. Genotyping was performed in real time with Mass ARRAY RT software version 3.0.0.4. Analysis was performed using mass array typer software version 3.4 (sequenom Inc., San Diego, CA, USA).

### 2.6 Statistical analysis

All statistical tests were analyzed using SPSS software for Windows version 23.0 (SPSS Inc., Chicago, IL). Each variant, and genotype distributions of the eight variants between IS and control were analyzed with a Chi‐squared test. A chi‐squared analysis was used to compare all categorical data. Normally distributed, continuous data were compared with a student's *t*‐test and expressed as mean ± standard deviation. The BH (Benjamini–Hocberg) method of FDR (False discovery Rate) was used to correct type I errors. The generalized multifactor dimensionality reduction (GMDR) beta v0.7 software package was used to analyze gene–gene interactions (βversion0.7, www.healthsystem.virginia.edu/internet/addiction‐genomics/Software), as previously described (Lou et al., 2007; Yi et al., 2019). GMDR software obtains the best model combination from multiple genes and behavioral indicators through the factor dimensionality reduction principle. The optimal model is obtained from the following results: (1) The model is meaningful only when the *p*‐value is less than 0.05; (2) The larger the testing balance accuracy is, the better the model effect is; (3) The closer the cross validation (CV) consistency is to 10, the better. The influence of high‐risk interactive genotypes on functional outcomes was investigated with multivariable logistic regression analysis, after adjusting for the main baseline variables related to each main variable in the univariate analysis (enter approach and probability of entry *p *< .2). A *p*‐value of less than .05 was considered a statistically significant difference (bilateral test).

## 3 RESULTS

### 3.1 Hardy–Weinberg equilibrium

The frequency distribution of the eight variants did not deviate from HWE (*p* > .05), indicating that gene frequency of the selected study population is representative of the gene distribution of the general population (Table 2).

**TABLE 2:** Hardy–Weinberg equilibrium of SNPs genotype in IS group and control group

SNPs	Genotype	IS group [*n*(%)]	*p *value	control group [*n*(%)]	*p *value
rs1799750	CC	249 (45.6)	.907	144 (41.1)	.555
	CT	241 (44.0)		157 (44.9)	
	TT	57 (10.4)		49(14.0)	
rs243865	CC	420 (76.7)	.896	274 (78.3)	.762
	CT	119 (21.8)		72(20.6)	
	TT	8 (1.5)		4 (1.1)	
Rs2285053	CC	310 (56.7)	.942	194 (55.4)	.546
	CT	204 (37.3)		136 (38.9)	
	TT	33 (0.60)		20 (5.7)	
Rs2241145	CC	133 (24.3)	.339	75 (21.4)	.423
	CG	262 (47.9)		182 (52.0)	
	GG	152 (27.8)		93(26.6)	
rs17576	AA	23 (4.2)	.177	29(8.3)	.061
	AG	204 (37.3)		119 (34.0)	
	GG	320 (58.5)		202(57.7)	
rs660599	AA	17 (3.1)	.922	4(1.2)	.061
	AG	157 (28.7)		102 (29.1)	
	GG	373 (68.2)		244 (69.7)	
rs2276109	CC	0 (0)	.453	1 (0.3)	.208
	CT	29 (5.3)		19 (5.4)	
	TT	518 (94.7)		330 (94.3)	
rs652438	CC	11 (2)	.926	2 (0.6)	.103
	CT	135 (24.7)		82(23.4)	
	TT	401 (73.3)		266 (76.0)	

### 3.2 Clinical characteristics of IS and controls

Demographic characteristics are summarized in Table 3. The proportion of hypertension and diabetes were higher in the IS group than in the control group. The TC, HDL, LDL, and HCY were higher in the IS group than in the control group. There was no significant difference between the two groups in terms of gender, age, smoking habits, alcohol consumption, or triglyceride levels (*p* > .05) (Table 3).

**TABLE 3:** Clinical characteristics in IS group and control group

Characteristics	IS group (*n *= 547)	Control group (*n *= 350)	*p* value
Gender (male) (n,%)	278/269	185/165	.552
Age (years, mean ± standard deviation)	62.21 ± 8.92	61.75 ± 8.67	.451
Cigarette smoking *(n*,％)	193 (35.3)	114 (32.5)	.404
Alcohol drinking (*n*,％)	117 (21.3)	79 (22.5)	.676
Hypertension (*n*,％)	405 (74.5)	168 (48.0)	<.005
Diabetes (*n*,％)	193 (35.3)	53 (15.1)	<.005
TG (mmol/L, mean ± standard deviation)	1.81 ± 1.38	1.79 ± 1.75	.858
TC (mmol/L, mean ± standard deviation)	5.39 ± 0.90	5.24± 1.18	.029
HDL (mmol/L, mean ± standard deviation)	1.30 ± 0.43	1.38 ± 0.49	.005
LDL (mmol/L, mean ± Standard Deviation)	3.79 ± 0.78	3.44 ± 0.93	<.005
HCY (mmol/L, mean ± standard deviation)	13.18 ± 7.58	10.49 ± 3.48	<.005

Abbreviations: HCY, Homocysteine; HDL, high density lipoprotein; LDL, low density lipoprotein; TC, total cholesterol; TG, triglycerides.

#### 3.2.1 Single‐locus analysis

The genotype distributions of *MMP‐1* (rs1799750), *MMP‐2* (rs23865, rs2285053, and rs2241145), *MMP‐9* (rs17576), and *MMP‐12* (rs660599, rs2276109, and rs652438) were compared between the IS and control group. The genotype distribution of rs17576 was statistically different between the two groups (*p* < .05). However, the genotype distribution of *MMP‐1* (rs1799750), *MMP‐2* (rs243865, rs2285053, and rs2241145), and *MMP‐12* (rs660599, rs2276109, and rs652438) were not statistically different between the two groups (*p* > .05) (Table 4).

**TABLE 4:** Genotype distribution comparison between IS and control

	IS group (*n *= 547)	Control group (*n *= 350)	*p* value
rs1799750			.194
CC	249 (45.6)	144 (41.1)	
CT	241 (44.0)	157 (44.9)	
TT	57 (10.4)	49(14.0)	
rs243865			.833
CC	420 (76.7)	274 (78.3)	
CT	119 (21.8)	72(20.6)	
TT	8 (1.5)	4 (1.1)	
rs2285053			.890
CC	310 (56.7)	194 (55.4)	
CT	204 (37.3)	136 (38.9)	
TT	33 (0.60)	20 (5.7)	
rs2241145			.448
CC	133 (24.3)	75 (21.4)	
CG	262 (47.9)	182 (52.0)	
GG	152 (27.8)	93(26.6)	
rs17576			.033
AA	23 (4.2)	29(8.3)	
AG	204 (37.3)	119 (34.0)	
GG	320 (58.5)	202(57.7)	
rs660599			.165
AA	17 (3.1)	4(1.2)	
AG	157 (28.7)	102 (29.1)	
GG	373 (68.2)	244 (69.7)	
rs2276109			.455
CC	0 (0)	1 (0.3)	
CT	29 (5.3)	19 (5.4)	
TT	518 (94.7)	330 (94.3)	
rs652438			.183
CC	11 (2)	2 (0.6)	
CT	135 (24.7)	82(23.4)	
TT	401 (73.3)	266 (76.0)	

### 3.3 Logistic analysis of risk factors of IS

From a univariate analysis, four SNPs candidates were selected for comparison with a logistic regression analysis between the IS and control groups for hypertension, diabetes, TC, HDL, LDL and HCY levels. Hypertension, diabetes, TC, LDL, HCY, and rs17576 were all independently associated with an increased risk of IS (Table 5). The AG genotype of rs17576 and the GG genotype of rs17576 were associated with a higher risk for IS with an OR and CI of 2.490 (1.251–4.959) and 2.494 (1.274–4.886), respectively (Table 5).

**TABLE 5:** Logistic analysis of risk factors of IS

Factor	0R	95% CI	*p* value^a^
Hypertension	2.788	2.027‐3.835	<.005
Diabetes	3.431	2.326‐5.063	<.005
TC	0.494	0.343‐0.711	<.005
LDL	3.613	2.374‐5.500	<.005
HCY	1.147	1.099‐1.197	<.005
rs17576			
AA			.025
AG	2.490	1.251‐4.959	.009
GG	2.494	1.274‐4.886	.008

^a^ Adjusted for hypertension, diabetes,TC, HDL, LDL, and homocysteine.

### 3.4 GMDR model for gene interactions

**FIGURE 1:**
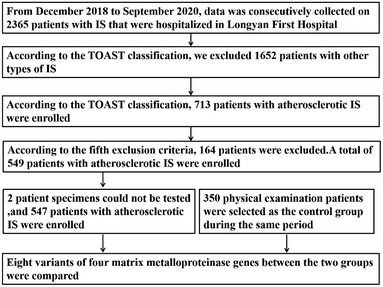
Research flow chart

The GMDR model of a gene–gene interaction between rs17576 and rs660599 was deemed the best. The cross‐validation consistency was 8/10 and the sign test was nine (*p* = 0.0107) (Table 6). The optimal model of interaction between rs17576 and rs660599 by GMDR is shown in Figure 1.

**TABLE 6:** GMDR model of gene interaction

Best model*	Training balanced accuracy	Testing balanced accuracy	Cross validation consistency	Sing test (*p*)
1	0.5255	0.4720	4/10	1 (.9990)
2,3	0.5517	0.5370	8/10	9 (.0107)
2,4,5	0.5693	0.4570	3/10	2(.9893)
1,2,4,5	0.6015	0.4861	5/10	5 (.6230)
1,2,3,4,5	0.6416	0.5077	10/10	6(.3770)
1,2,3,4,5,6	0.6726	0.4935	10/10	5(.6230)
1,2,3,4,5,6,7	0.6830	0.4915	10/10	4(.8281)
1,2,3,4,5, 6,7,8	0.6835	0.4911	10/10	4(.8281)

* The numbers 1–8 represent rs2241145, rs17576, rs660599, rs2285053, rs1799750, rs243865, rs652438, rs2276109.

### 3.5 Interaction analysis for rs17576 and rs660599 using logistic regression

**FIGURE 2:**
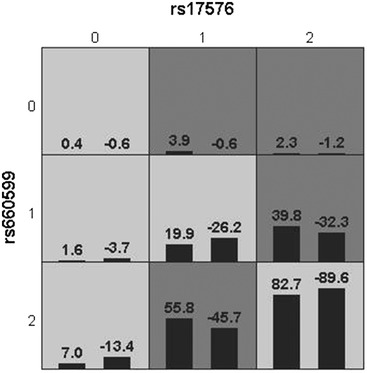
Risk of 9 different combinations of rs17576 and rs660599 genotypes. High‐risk cells are indicated by dark color, low‐risk cells are indicated by light color. The high‐risk interaction genotype was assigned as one, and low‐risk interaction genotype was assigned as zero in multivariable logistic regression analysis. The numbers 0–2 represent rs17576 AA,AG and GG, and rs660599 AA,AG and GG, respectively

According to nine different combinations of rs17576 and rs660599 genotypes in Figure 1, they are divided into high risk and low risk, high risk combinations are indicated by darker coloring (1), low risk combination are indicated by lighter coloring (0) (Figure 2) (assignment: high risk = 1; low risk = 0). After adjusting the factors of hypertension, diabetes, TC, HDL, LDL, and HCY by multivariate logistic regression analysis, it was found that having the combination of rs17576 and rs660599 SNPs was correlated with higher risk for IS with an OR of 1.568 and a CI of 1.152–2.135 (Table 7).

**TABLE 7:** Multivariate logistic regression analysis of gene‐gene interaction in IS

*Factor*	0R	95% CI	*p* value^a^
Hypertension	2.707	1.979‐3.704	<.005
Diabetes	3.096	2.120‐4.522	<.005
TC	0.497	0.348‐0.709	<.005
LDL	3.572	2.366‐5.395	<.005
Homocysteine	1.141	1.094‐1.190	<.005
rs17576 interaction with rs660599	1.568	1.152‐2.135	.004

^a^ Adjusted for hypertension, diabetes, TC, HDL, LDL, and homocysteine.

## 4 DISCUSSION

This case‐control study is one conducted on the Han Hakka population that aimed to investigate the association between *MMP‐1* (rs1799750), *MMP‐2* (rs243865, rs2285053, and rs2241145), *MMP‐9* (rs17576), and *MMP‐12* (rs660599, rs2276109, and rs652438) genotypes. It is the first one conducted on SNP–SNP interactions among *MMP* genes and the risk of IS in the Han Hakka population in Western Fujian, China. Haplotypic variation in *MMP‐1* (rs1799750), *MMP‐2* (rs243865, rs2285053, and rs2241145), and *MMP‐12* (rs660599, rs2276109, and rs652438) were not associated with a higher risk for IS, however, haplotypic variation in *MMP‐9* rs17576 correlated with an increased risk of IS. GMDR analysis revealed that having both rs17576 and rs660599 SNPs gives rise to an increased risk of IS as well.

Several studies have reported no association between *MMP* *‐1, 2*, or *12* polymorphisms and ischemic stroke risk, which is consistent with this study. Chehaibi et al. showed that there was no significant correlation between the *MMP‐1*‐1607G/2G gene polymorphism and atherosclerosis in Tunisian patients (Chehaibi et al., 2014). Yunhua Hao et al. revealed that *MMP‐2* rs243865 was not related to cerebral infarction in the Chinese Han population (Hao et al., 2015). Simultaneously, Yeon Jung Kim et al. demonstrated that *MMP‐2* rs243865 was not related to cerebral infarction (Kim et al., 2020). Furthermore, Marc Fatar et al. showed that there is an association of the *MMP‐2* gene (rs1030868, rs2241145, rs2287074, rs2287076, and rs7201) with the development of lacunar stroke, but no association of *MMP‐2* with other stroke subtypes (Fatar et al., 2008). Weiling Li et al. showed that *MMP‐12* rs2276109 was not associated with atherosclerosis (Li et al., 2012). This study found that *MMP‐9* rs17576 was related to IS. Mehrdad Sheikhvatan et al. also showed that *MMP‐9*‐C1562T and *MMP‐9* rs17576 gene polymorphisms were related to coronary atherosclerosis (Sheikhvatan et al., 2018). Additionally, Xianjing Feng et al. reveled *MMP‐9* rs17576 may be associated with the risk of intracranial atherosclerotic stenosis (Feng et al., 2021). Nevertheless, Alexey Polonikov et al. found that *MMP‐9* rs17576 did not increase the risk of cerebral infarction alone but increased the risk of cerebral infarction as a part of a gene network (Polonikov et al., 2019). However, there are many studies that are inconsistent with these findings (Djurić et al., 2012; Manso et al., 2010; Traylor et al., 2014). The diversity of these outcomes may be due to ethnic differences, study design, and sample size as well as fortuity. As a matter of fact, it is likely that there are multiple variations in the pathogenesis of cerebral infarction, each with a slight or potentially undetectable effect (Schork et al., 2009). Due to gene–gene and gene–environment interactions, linkage analyses are commonly used for single‐gene disease studies and may not be suitable for genetic studies of stroke.

GMDR is a tool used to study gene–gene interactions and has become a hot tool in gene interaction research (Lou et al., 2007). Nevertheless, significant observations were made in this study using the GMDR method. Through GMDR study, it was found that the interaction between *MMP‐9* rs17576 and *MMP‐12* rs660599 increases the risk of IS by 1.568‐fold. This result suggests the interaction of these two gene polymorphisms may play a key role in genetic susceptibility to IS. Recent genome‐wide association studies have shown the presence of common genetic variants increases the risk of ischemic stroke, but most of the research has focused only on single genes. These findings add to the evidence that genes–gene interactions can increase the risk of complex diseases, such as ischemic stroke. The combinatorial analysis used in this study may be helpful in the elucidation of complex genetic risk factors for common diseases like IS.

*MMP‐9* is a 92‐kDa protein that belongs to a family of zinc‐ and calcium‐dependent endopeptidases (Fenhalls et al., 1999; Pourmotabbed et al., 1994) and plays a key role in all stages of atherosclerosis through monocyte recruitment influence, ECM degradation, endothelial cell migration, and activation of vascular smooth muscle cells (Blankenberg et al., 2003; Hirose et al., 2008; Ye, 2006). *MMP‐9* gene polymorphisms encode and regulate the transcription of the *MMP‐9* protein, and correlate with the concentration of *MMP‐9* in plasma (Luizon et al., 2016). Genetic polymorphisms located in promoter regions of *MMP* genes can lead to increased gene expression and may be associated with susceptibility to various diseases (Blankenberg et al., 2003). *MMP‐9* R279Q polymorphism is a glutamine‐arginine substitution in the catalytic domain of *MMP‐9*, which may affect substrate binding. Although the interaction between *MMP‐9* rs17576 and *MMP‐12* rs660599 can increase the risk of IS, the interactions between the two gene variants are unclear. Previous studies have shown that both *MMP‐9* and *MMP‐12* participate in the process of monocyte recruitment and ECM degradation (Pérez‐Rial et al., 2013)^.^ Furthermore, *MMP‐9* and *MMP‐12* cause n‐cadherin shedding and thereby beta‐catenin signaling, with subsequent vascular smooth muscle cell proliferation (Dwivedi et al., 2009)^.^ Moreover, joint effects or haplotypes of *MMP* polymorphisms are stronger than the individual effect of each polymorphism. *MMPs* serve as target genes for gene regulatory networks driving molecular and cellular pathways related to a multistep pathogenesis of cerebrovascular disease (Polonikov et al., 2019).

The results of this study once again indicate that the *MMP‐9* rs17576 polymorphism is significantly associated with atherosclerotic ischemic stroke. This study provides a theoretical basis for future research on *MMP* gene polymorphisms and their role in ischemic stroke. Previous studies mainly focused on a single gene polymorphism, with only a few studies on gene‐gene interactions. The main discovery of this study is that interaction between *MMP‐9* rs17576 and *MMP‐12* rs660599 is associated with increased risk of ischemic stroke. These results provide a theoretical basis for the role of gene–gene interaction in ischemic stroke.

Despite these interesting findings, there are of course limitations to this study. First, these results need to be verified with larger, multicenter studies because of limited sample size and the general nature of single center studies. It is planned to cooperate with other local hospitals to expand the sample size for relevant content research. Second, only a few *MMP* polymorphisms were studied, many important polymorphic genes of *MMPs* and their interactions were excluded in the study, begging the need for further research to be performed. Third, only patients with atherothrombosis were enrolled in this study, the other subtypes of stroke and transient ischemic attacks were excluded. The association of an *MMP9* SNP or *MMP* SNP‐SNP interaction with other subtypes of stroke or transient ischemic attacks is unknown. Therefore, further studies are necessary.

## 5 CONCLUSION

This study shows the *MMP‐9* rs17576 gene polymorphism is associated with increased IS risk while the other seven gene polymorphisms studied were not significantly associated with increased risk for IS in the Han Hakka population. Simultaneously, it was revealed, through GMDR analysis, that interaction between *MMP‐9* rs17576 *and MMP‐12* rs660599 is associated with increased IS risk in the Han Hakka population.

## CONFLICT OF INTEREST

The authors declare no conflicts of interest.

### PEER REVIEW

The peer review history for this article is available at https://publons.com/publon/10.1002/brb3.2473

## Data Availability

All data generated during the project will be made available upon the request from the corresponding author. There are no security, licensing, or ethical issues related to these data.
